# The Uterotonic Screening of the Root Extract of *Azanza garckeana* (Malvaceae) on Isolated Wistar Rat Uterine Smooth Muscles

**DOI:** 10.1155/2020/8873180

**Published:** 2020-11-27

**Authors:** Alfred Chanda, Freddie Simwinga, Patrick Kaonga, Angela Gono-Bwalya, Lavina Prashar

**Affiliations:** ^1^The University of Zambia, School of Medicine, Department of Physiological Sciences, Lusaka, Zambia; ^2^The University of Zambia, School of Public Health, Department of Epidemiology and Biostatistics, Lusaka, Zambia; ^3^The University of Zambia, School of Health Sciences, Department of Pharmacy, Lusaka, Zambia

## Abstract

**Introduction:**

*Azanza garckeana* (F.Hoffm.) Exell and Hillc. (family: Malvalceae) is traditionally used to induce or accelerate labour in pregnant women in Chongwe, Zambia, and the plant part which is commonly used are the roots.

**Aim:**

The aim of this study was to screen *Azanza garckeana* crude extracts for uterotonic activity on isolated Wistar rat uterine smooth muscles. The likely mechanism of action for the plant extract was also investigated.

**Materials and Methods:**

Fresh leaves and roots of the plant were collected and identified by a botanist at the University of Zambia. The methanol and cold root aqueous extracts were prepared by continuous maceration while the hot aqueous root extract was extracted using the Soxhlet method. The crude extracts of the plant were screened for uterotonic activity using uterine smooth muscles isolated from estrogenised adult nongravid female Wistar rats weighing between 160 g and 200 g. The activity of the plant was also evaluated in the presence of antagonists and tocolytic agents to determine the likely mechanism of action.

**Results:**

The hot aqueous root crude extract (22.26%) had the highest yield followed by the cold aqueous (11.32%) and methanol extracts (6.26%), respectively. The methanol crude root extract demonstrated the highest potency (EC_50_ = 1.28 × 10^−2^ mg/ml; 95% CI 6.418 × 10^−3^ to 2.564 × 10^−2^; *p*=0.0001), while the cold aqueous extract was the most efficacious. Salbutamol and nifedipine significantly blocked the uterotonic activity of the extract.

**Conclusions:**

This study provides scientific evidence on the uterotonic activity of *Azanza garckeana* with myometrial calcium mobilization as the possible mechanism of action.

## 1. Introduction

Screening of herbal medicines according to their traditional use is essential and useful as it is documented that 25% of prescription drugs are obtained from medicinal plants [1]. It is estimated that there are approximately 250,000 higher plant species worldwide, and of these, only a few have been screened for pharmacological activity according to their traditional use [2]. Indigenous varieties of plants from various families are used by pregnant mothers in rural areas and low-income populations of sub-Saharan Africa for their uterotonic potential [3].

Approximately 80% of pregnant women in African countries use herbal medicine to induce or accelerate labour [4]. Several plants have been screened for their uterotonic activity using *in vitro* methods with positive results [5]. In Zambia, about 30–32% of pregnant women use herbal medicine to induce or accelerate labour [6, 7]. One such herbal medicine is Mukole plant which is scientifically known as *Azanza garckeana* (F.Hoffm.) Exell and Hillc. from the Malvalceae family (http://www.theplantlist.org). The questionnaire structured cross-sectional study by Maluma et al. [7] to determine the prevalence and factors associated with the use of traditional medicines in Lusaka Province, Zambia (in Chawama urban and Chongwe rural communities), found that Mukole plant (*Azanza garckeana*) was one of the commonly used uterotonic plants. Mukole is a vernacular name used by the Nsenga people from the Eastern Province of Zambia to describe the plant [8]. *Azanza garckeana* is also used to induce labour in pregnant women in Tanzania [9].

We, therefore, set out to screen uterotonic activity and potential mechanism of action of *Azanza garckeana* on isolated nongravid estrogenised Wistar rat uterine smooth muscles. The findings of this study provide scientific evidence to the uterotonic activity of *Azanza garckeana*.

## 2. Methodology

### 2.1. Study Design

This was an experimental study designed by pretreating adult nongravid female virgin Wistar rats with 0.2 mg/kg diethylstilbestrol 24 hours before the experiment. On the day of the experiment, the rats were sacrificed, and uterus was isolated, cut into longitudinal strips, and suspended in a 50 ml organ bath (AD instruments). Baseline uterotonic contractions and oxytocin were used as negative and positive controls, respectively. Each set of the experiment was done in triplicate.

### 2.2. Data Collection and Analysis

In this study, numerical data was collected using data collection tables and LabTutor software. Data were collected and entered into Microsoft Excel and exported to STATA version 13. Data were presented as means and standard deviations. One-way ANOVA and Bonferroni post-hoc tests were done in STATA. Bar charts and dose-response curves were done using Graphpad Prism version 5.00 for Windows (San Diego, California, USA).

### 2.3. Study Site

The study was conducted at the Pharmacology Laboratory, Department of Physiological Sciences, School of Medicine, University of Zambia.

### 2.4. *Azanza garckeana* Plant Collection and Identification

Fresh leaves and roots of *Azanza garckeana* plant were collected from a location 15°16′17.3′ south and 28°45′53.0′ east in Kapete Ward, Chongwe, Lusaka Province, Zambia, with the aid of a local herbalist. The plant voucher specimen (accession number 22209) was identified by a botanist at the University of Zambia Herbarium (UZL).

### 2.5. *Azanza garckeana* Plant Extraction

*Azanza garckeana* roots were cut in small pieces, shade dried in a well-ventilated area for 14 days, and ground to powder using a blender (1.75 litres Satin Russell Hobbs Blender, India). The powder of the root material was stored in airtight Ziploc plastics until required.

### 2.6. Preparation of the Methanol and Cold Aqueous Crude Extracts

The crushed root material (50 g) was extracted using 99.5% methanol (500 ml) and distilled water (500 ml) by continuous maceration using a magnetic stirrer for 72 hours. The extracts were then filtered with Whitman No.1 filter paper (Merck, Germany). The crude extracts were then dried to obtain a powder whose yield was calculated. The extracts were then parked in airtight Ziploc plastics and stored in a refrigerator at 4°C until required.

### 2.7. Preparation of the Hot Aqueous Crude Extract

Distilled water (1000 ml) was added to a round bottom flask attached to a Soxhlet extractor and condenser on a heating isomantle. The crushed plant root material (100 g) was loaded into the thimble, which was placed inside the Soxhlet extractor. The sidearm was lagged with glass wool. The solvent was heated using a heating isomantle and was allowed to begin to evaporate, moving through the apparatus to the condenser. The condensate then dripped into the reservoir containing the thimble. Once the level of solvent reaches the siphon, it was poured back into the flask, and the cycle was repeated. After completion of the process, the extract was dried to powder.

### 2.8. Experimental Animals (Specimen)

The animals were sourced and housed in the animal house at the University of Zambia, School of Medicine, Department of Physiological Sciences. The female Wistar rats were separated from the male Wistar rats as soon as the sex was identified. Rats were kept in plastic cages (1 cage per rat) at room temperature and on a 12-hour light/dark cycle with access to pellet food and water ad libitum. The adult female virgin Wistar rats weighing 150 to 200 g and aged 5 to 6 months old were used for the experiment.

### 2.9. Standard Drugs Used for the Experiment

The drugs that were used in this study are as follows:

  Diethylstilbestrol injection (Kunj Pharma pvt., Ltd., India) was used to estrogenise the nongravid Wistar rats
  Oxytocin injection (Mylan Health pvt., Ltd., Australia) was used as a standard uterotonic drug
  Cyproheptadine 4 mg tablets (Shalina Healthcare, India) were used to block histamine 1 receptors of the uterine smooth muscles
  Salbutamol 4 mg tablets (Lincon Pharm Ltd., India) were used to stimulate beta-2 receptors of the uterine smooth muscles
  Atropine injection (Actiza Pharma pvt., Ltd.) was used to block the muscarinic receptor of the uterine smooth muscles
  Indomethacin 25 mg capsules (JNB Pharm pvt., Ltd, India) were used to inhibit the cyclooxygenase (COX) enzymes of the uterine smooth muscles
  Nifedipine 20 mg tablets (Denk Pharma, Germany) were used to block the L-type calcium channel of the uterine smooth muscles

### 2.10. Mounting of Isolated Wistar Rat Uterus in the Organ Bath

In preparation for uterus isolation, the female Wistar rats were estrogenised with 0.2 mg/kg diethylstilbestrol (DES) subcutaneously 24 hours before the experiment. DES was reconstituted with ethanol/water (1 : 1) solution before drug administration. DES is a synthetic estrogenic drug that is more potent than natural estrogen which was used to induce oestrus; the drug can induce the formation of gap junctions in the endometrial cells of the uterus, which in turn promote thickening of the endometrial layer of the uterine smooth muscles and allow it to work as a single unit. On the day of the experiment, the rats were euthanized by cervical dislocation. The uterine horns were dissected out, cleansed out of excess fat and connective tissues, and cut into longitudinal strips. The uterine strips were suspended in the 50 ml organ bath containing De Jalon's physiological solution made up of 9 g/l sodium chloride (NaCl), 0.5 g/l sodium hydrogen carbonate (NaHCO3), 0.5 g/l D glucose, 0.402 g/l potassium chloride (KCL), and 0.08 g/l hydrated calcium chloride (CaCl_2_ × 2H_2_O). The organ bath was maintained at 37°C and aerated with a mixture of 95% oxygen (O_2_) in 5% carbon dioxide (CO_2_). The strips were connected to the force transducers which were in turn connected to PowerLab and LabTutor (AD Instruments). The tissue tension was adjusted using the transducers to the resting uterine smooth muscle contractions of 5 mN, and then, the force of contraction was zeroed (0 mN) using the PowerLab. The suspended uterine smooth muscle strips connected to the transducers were allowed to equilibrate for at least 30 minutes to obtain the baseline uterus contractions before the samples were applied to the organ bath where the uterine smooth muscle strips were suspended. The tissue activity was monitored and observed using the LabTutor, before and after an intervention.

### 2.11. Uterotonic Evaluation

Noncumulative doubling concentrations of *Azanza garckeana* extract and of the standard drugs were added one at a time to the De Jalon's physiological solution in the organ bath where the uterine strip was suspended. Each concentration was allowed to act for 10 minutes during which the amplitude of contraction was measured. The experiment was done in triplicate (*n* = 3) for each of the concentrations. The extract and drugs were reconstituted with De Jalon's physiological solution. The amplitude of uterine smooth muscle contractions was recorded using a LabTutor computer software.

The uterotonic activity of *Azanza garckeana* crude extracts was first evaluated with the initial final bath concentration of 1.6 × 10^−4^ mg/ml. Then, subsequent concentrations were doubled until the maximum sustained contractions were obtained at the final bath concentration of 6.55 × 10^−1^ mg/ml. The 13 observed amplitude of contractions produced by the doubling noncumulative bath concentrations of the extracts were subjected to one-way ANOVA. The Bonferroni post-hoc statistical test was done on 13 different noncumulative final bath concentrations to test at which bath concentration the uterotonic activity significantly started.

### 2.12. Mechanism of Action of the Root Extract of *Azanza garckeana*

The uterotonic activity of *Azanza garckeana* methanol extract was evaluated in the presence of antagonists and tocolytic drugs. The uterine smooth muscle contractions produced by the methanol crude extract (1.28 × 10^−2^ mg/ml) was evaluated in the presence of antagonist and tocolytic drugs whose concentration values were obtained from previous studies [10–12]. The concentrations of antagonists and tocolytic agents used in the study were 2.7 ml (1.48 mg/ml) for cyproheptadine, a histamine H1 blocker, 5 ml (8.00 × 10^−1^ mg/ml) for salbutamol, a physiological antagonist, 1 ml (6.00 × 10^−1^ mg/ml) for atropine, a muscarinic receptor blocker, 5 ml (10 mg/ml) indomethacin, a cyclooxygenase inhibitor, and 5.8 ml (3.46 × 10^−1^ mg/ml) for nifedipine, the L-type calcium channel blocker. Antagonists and tocolytic agents were allowed to act for 10 minutes before the methanol extract was applied to the organ bath.

### 2.13. Ethical Considerations

In this study, all animal rights were respected [13, 14]. The animals were treated well and were given access to standard nutrition, water, and environment at the animal house manned by a trained animal attendant from the Department of Physiological Sciences, School of Medicine, University of Zambia. The animals were euthanized by cervical dislocation before the isolation of the uterus. Approval was sought and obtained from the University of Zambia Biomedical Research Ethics Committee (UNZABREC) before the study was conducted (animal ethics approval number: 006-12-18). Permission to carry out the study was also granted by the National Health Research Authority (NHRA).

## 3. Results and Discussion

### 3.1. Experimental Animal Models

In this study, the screening of the uterotonic activity of the root methanol crude extract of *Azanza garckeana* was done on the uterine smooth muscles isolated from estrogenised adult healthy virgin female Wistar rats. Wistar rats were used in this study because their genetic, biological, and behavioural characteristics closely resemble humans [15]. Most of the Wistar rats used in the study (40%) were aged five months, 27% were aged six months. This study considered age as an important characteristic of the animal model used and included mature Wistar rats, which are suited for the screening of the uterotonic activity of *Azanza garckeana* [16]. The rats used weighed from 150 to 200 g which is the same as other similar studies [17–23].

### 3.2. Description of *Azanza garckeana* Crude Extracts

**Figure 1:**
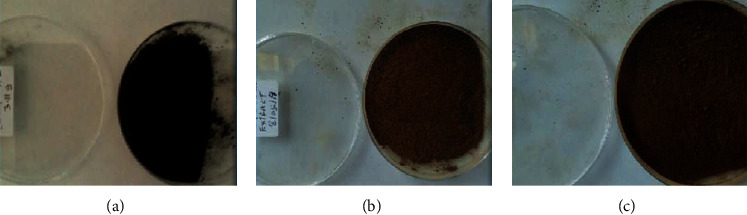
*Azanza garckeana* crude root extracts. (a) Methanol extract. (b) Hot aqueous extract. (c) Cold aqueous extract.

The methanol solvent yielded a dark brownish-black extract while the cold and hot water solvents yielded a brownish extract (Figure 1).

### 3.3. Percentage Yield of *Azanza garckeana* Crude Extracts

The percentage (%) yield of the *Azanza garckeana* crude extracts was calculated using the following formula:

(1) $$\begin{array}{c} \%\text{ Yield}=\frac{\text{weight of crude root extract}}{\text{weight of plant powder}}\times 100. \end{array}$$

The hot aqueous solvent yielded the highest crude extract (22.26%) while the cold aqueous extract (11.32%) and methanol extract (6.26%) were second and third, respectively. This may suggest that the hot aqueous extract may have the highest yield of phytochemicals. Other studies on the plant have reported on the percentage yield of other parts of *Azanza garckeana* and not the root. One study looked at the effect of extraction solvents on the phytochemical yield of the pulp and shaft of *Azanza garckeana* and reported the methanol extract to have the highest yield [24].

### 3.4. Determining the Crude Root Extract with the Highest Uterotonic Activity

#### 3.4.1. Uterotonic Activity of Oxytocin

The uterotonic activity produced by the 13 doubling noncumulative concentrations of oxytocin was statistically significant by one-way ANOVA (*p* < 0.0001). Bonferroni post-hoc test showed that the significant uterotonic activity started at the concentration of 2.00 × 10^−8^ mg/ml. The concentration-response curve for oxytocin gave the best fit sigmoidal slope with the EC_50_ at 1.17 × 10^−7^ mg/ml (95% CI 3.18 × 10^−8^ to 4.32 × 10^−7^ mg/ml; *p*=0.0001) and the Emax at 4.10 × 10^−5^ mg/ml. The amplitude of uterine smooth muscle contraction for oxytocin increased linearly with an increase in concentration and reached a plateau at 1.28 × 10^−6^ mg/ml.

Oxytocin was used as a positive control for uterotonic activity because it is the drug of choice used to induce or accelerate labour and also in the management of postpartum hemorrhage.

#### 3.4.2. The Uterotonic Activity of Three Crude Extracts of *Azanza garckeana*

The amplitude of uterine smooth muscle contractions produced by the three crude extracts of *Azanza garckeana* were all found to be statistically significant using one-way ANOVA. Bonferroni post-hoc statistical test showed that the hot aqueous extract significant uterotonic activity started at the lowest concentration of 1.02 × 10^−2^ mg/ml (*p* < 0.0001^*∗∗*^), followed by the methanol extract at 2.05 × 10^−2^ mg/ml (*p* < 0.0001^*∗∗*^) and the cold aqueous extract at 3.28 × 10^−1^ mg/ml (*p* < 0.0001^*∗∗*^). All the crude extracts demonstrated the Emax at the concentration of 6.55 × 10^−1^ mg/ml above which there was no further increase in response.

The cold aqueous extract showed the highest efficacy (28 mN), followed by the methanol extract (20.75 mN) and hot aqueous extract (19.06 mN), respectively.

**Figure 2:**
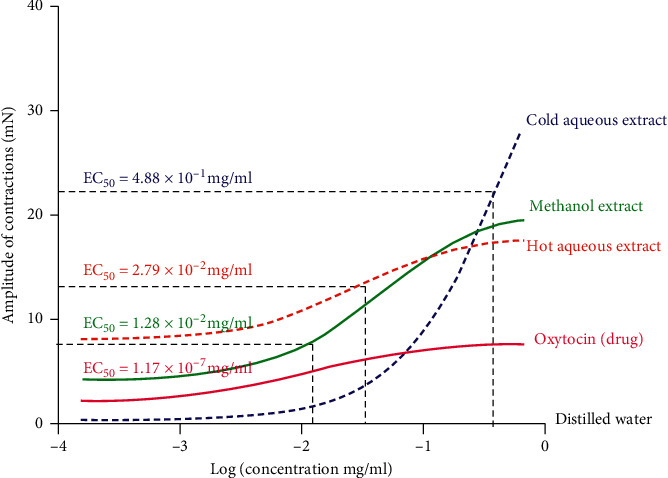
Concentration-dependent contraction of the uterine smooth muscle strips to *Azanza garckeana* crude root extracts. Points represent means_S.E.M. for the number of experiments (*n* = 3). Distilled water, negative control; oxytocin, positive control.

The *Azanza garckeana* methanol crude extract was the most potent (EC_50_ 1.28 × 10^−2^ mg/ml; 95% CI 6.418 × 10^−3^ to 2.564 × 10^−2^; *p*=0.0001) followed by the hot aqueous (EC_50_ 2.79 × 10^−2^ mg/ml; 95% CI 4.882 × 10^−3^ to 1.597 × 10^−1^, *p* < 0.0001^*∗∗*^) and cold aqueous extracts, respectively (EC_50_ 4.88 × 10^−1^ mg/ml; 95% CI 2.20 × 10^−1^ to 1.08, *p* < 0.0001^*∗∗*^) (Figure 2).

The potency of all the three crude extracts was statistically different from distilled water (methanol extract *p* < 0.001^*∗*^, hot aqueous extract *p* < 0.001^*∗*^, cold aqueous extract *p* < 0.001^*∗*^) and from each other (*p* < 0.0001^*∗∗*^). The methanol extract of *Azanza garckeana* demonstrated the highest potency suggesting that the highly potent compound with uterotonic activity can be obtained from this extract.

The cold aqueous extract of *Azanza garckeana* was less potent (EC_50_ = 4.88 × 10^−1^ mg/ml) than the hot aqueous extract (EC_50_ = 2.79 × 10^−2^ mg/ml) suggesting that the hot aqueous extraction method (Soxhlet) may be better than that of the cold aqueous extraction method (maceration). Although the methanolic extract had the highest potency, the cold aqueous extract demonstrated the highest efficacy on uterine smooth muscles. The high amplitude of contraction produced by the cold aqueous crude root extract of *Azanza garckeana* may suggest the plant's contribution to the prevalence of uterine rupture experienced by pregnant women who take uterotonic herbs for inducing or accelerating labour [25, 26]. The uterotonic activity of all the crude extracts reduced to baseline after the maximum concentration despite any further increment in the concentration of the extract. The downward reduction in activity after the Emax for all the extracts may suggest the extracts to have a biphasic effect of uterine smooth muscle contraction and relaxation.

Solvent types are essential in plant crude extraction methods and each method is unique to the plants. The evaluation of various extraction methods for the plant is important as it provides suitable extraction methods to be used in future studies [27]. The findings of this study provide scientific evidence on the solvent extraction method with appropriate uterotonic compound composition to be used in the identification of the major uterotonic compound from the root of *Azanza garckeana* [28, 29].

### 3.5. Mechanism of Action of *Azanza garckeana*

**Figure 3:**
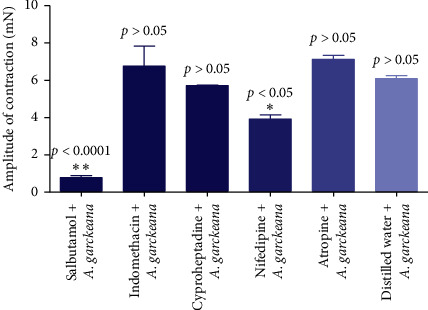
Uterotonic activity of *Azanza garckeana* in the presence of antagonists and tocolytic drugs. Values expressed as mean ± SD (*n* = 3 per experiment). One-way ANOVA to compare the amplitude of contraction produced by *Azanza garckeana* methanol crude root extract in the presence of distilled water (control) to its amplitude of contraction in the presence of antagonist and tocolytic agents (*p* < 0.05^*∗*^, *p* < 0.001^*∗∗*^).

One-way ANOVA test showed a significant reduction in the *Azanza garckeana* extract uterotonic activity in the presence of antagonist and tocolytic agents (*p* < 0.0001). There was a significant reduction in the amplitude of contraction produced by *Azanza garckeana* methanol crude root extract in the presence of salbutamol (*p* < 0.0001) and nifedipine (*p*=0.0048). The reduction in the amplitude of contraction produced by indomethacin (*p*=1.000), cyproheptadine (*p*=1.000), and atropine (*p*=1.000) was not statistically significant (Figure 3).

Salbutamol reduced the amplitude of contraction produced by the methanol crude extract from 6.09 mN to 0.78 mN. Nifedipine significantly reduced the uterotonic activity of *Azanza garckeana* methanol extract from 6.09 mN to 3.92 mN. The significant reduction in activity caused by the two drugs may suggest the plant to produce its effect via calcium mobilization in the myometrium. However, further studies need to be done to ascertain this assumption on the likely mechanism of action for the plant.

## 4. Conclusion

The study found that *Azanza garckeana* crude root extract possesses uterotonic potential. The methanol crude root extract of the plant was the most potent as compared to other extracts. The efficacy of the plant was significantly reduced by salbutamol and nifedipine, suggesting the uterotonic activity of the plant to be produced through calcium mobilization in the myometrium.

Further pharmacological and toxicological studies need to be done on the plant to determine the safety profile of the plant and to identify the constituents responsible for the reported activity. Further studies also need to be done to confirm the likely mechanism of action for the plant.

## Data Availability

The data used to support the findings of this study are available from the corresponding author upon request.

## Supplementary Materials

Supplementary Materials Table 1: uterotonic activity of *Azanza garckeana* crude extracts on uterine smooth muscle.
