# Supplementary material for: The Uterotonic Screening of the Root Extract of Azanza garckeana (Malvaceae) on Isolated Wistar Rat Uterine Smooth Muscles
Source: Evid Based Complement Alternat Med. 2020 Nov 27;2020:8873180. doi: 10.1155/2020/8873180 (PMC7744173; doi:10.1155/2020/8873180)
Supplement: Supplementary Materials — Table 1: uterotonic activity of Azanza garckeana crude extracts on uterine smooth muscle. [file 8873180.f1.docx]

**SUMPLIMENTARY INFORMATION**

**Table 1: Uterotonic activity of Azanza garckeana crude extracts on uterine smooth muscle**

|  |  |  | **METHANOL CRUDE EXTRACT** | **HOT AQUEOUS EXTRACT** | **COLD AQUEOUS EXTRACT** |
| --- | --- | --- | --- | --- | --- |
| **No** | **Concentration (mg/ml)** | **Log dose (mg/ml)** | **Amplitude of contraction (mN)** | **Amplitude of contraction (mN)** | **Amplitude of contraction (mN)** |
| 1 | 1.60 x 10^-4^ | -3.8 | 0.00 ± 0.00 | 0.00 ± 0.00 | 0.00 ± 0.00 |
| 2 | 3.20 x 10^-4^ | -3.49 | 1.26 ± 0.22 | 8.05 ± 0.70 | 0.14 ± 0.25 |
| 3 | 6.40 x 10^-4^ | -3.19 | 1.81 ± 0.31 | 8.92 ± 0.78 | 0.27 ± 0.46 |
| 4 | 1.28 x 10^-3^ | -2.89 | 6.60 ± 0.47 | 11.69 ± 0.35 | 0.59 ± 0.02 |
| 5 | 2.56 x 10^-3^ | -2.59 | 7.55 ± 0.35 | 12.04 ± 0.62 | 0.84 ± 0.14 |
| 6 | 5.12 x 10^-3^ | -2.29 | 8.66 ± 0.75 | 13.47 ± 1.00 | 1.22 ± 0.21 |
| 7 | 1.02 x 10^-2^ | -1.99 | 9.00 ± 0.50 | 14.23 ± 0.20** | 1.64 ± 0.28 |
| 8 | 2.05 x 10^-2^ | -1.69 | 10.10 ± 0.22* | 14.44 ± 0.15** | 3.31 ± 0.29 |
| 9 | 4.10 x 10^-2^ | -1.39 | 13.27 ± 0.28** | 15.14 ± 0.22** | 3.97 ± 0.49 |
| 10 | 8.19 x 10^-2^ | -1.09 | 14.49 ± 0.35** | 15.62 ± 0.26** | 6.28 ± 0.61 |
| 11 | 1.64 x 10^-1^ | -0.79 | 16.81 ± 0.36** | 16.31 ± 0.22** | 11.44 ± 1.62 |
| 12 | 3.28 x 10^-1^ | -0.48 | 17.25 ± 0.22** | 18.59 ± 0.18** | 22.74 ± 0.65** |
| 13 | 6.55 x 10^-1^ | -0.18 | 20.75 ± 0.12** | 19.06 ± 0.10** | 28.00 ± 0.58** |

**Key: mN= milinewtons**

The table above shows values expressed in mean ***±*** SD (n=3 per experiment), analysis was done using one-way ANOVA which was followed by Bonferroni post hoc test to compare the amplitude of contraction produced by distilled water (control) to the amplitude of contraction produced by the 13 non-cumulative bath concentrations of *Azanza garckeana* crude root extract (p>0.05, p<0.05*, p<0.001**).
